# Plasma Metabolome Profiling by High-Performance Chemical Isotope-Labelling LC-MS after Acute and Medium-Term Intervention with Golden Berry Fruit (*Physalis peruviana* L.), Confirming Its Impact on Insulin-Associated Signaling Pathways

**DOI:** 10.3390/nu13093125

**Published:** 2021-09-07

**Authors:** Fabrice Vaillant, Vanesa Corrales-Agudelo, Natalia Moreno-Castellanos, Alberto Ángel-Martín, Juan Camilo Henao-Rojas, Katalina Muñoz-Durango, Patrick Poucheret

**Affiliations:** 1 Corporación Colombiana de Investigación Agropecuaria–Agrosavia, Centro de Investigación La Selva, Kilómetro 7, Vía a Las Palmas, Vereda Llanogrande, Rionegro 054048, Colombia; jhenao@agrosavia.co; 2 French Agricultural Research Centre for International Development (CIRAD), UMR Qualisud, 34398 Montpellier, France; 3 Joint Research Unit—UMR Qualisud, CIRAD, University Montpellier, Avenue Agropolis, CEDEX 5, 34398 Montpellier, France; patrick.poucheret@umontpellier.fr; 4 Vidarium-Nutrition, Health and Wellness Research Center, Nutresa Business Group, Calle 8 sur No. 50-67, Medellin 050023, Colombia; vcorrales@serviciosnutresa.com (V.C.-A.); kmunoz@serviciosnutresa.com (K.M.-D.); 5 CINTROP, Facultad de Salud, Universidad Industrial de Santander, Cra. 32 29-31, Bucaramanga 680002, Colombia; nrmorcas@uis.edu.co; 6 OENEC, Escuela de Nutrición y Dietética, Facultad de Salud, Universidad Industrial de Santander, Cra. 32 29-31, Bucaramanga 680002, Colombia; angelmar@uis.edu.co

**Keywords:** metabolomic, *Physalis peruviana*, nutritional intervention, insulin

## Abstract

Purpose: Golden berry (*Physalis peruviana* L.) is an exotic fruit exported from Colombia to different countries around the world. A review of the literature tends to demonstrate a hypoglycaemic effect with an improvement in insulin sensitivity after oral ingestion of fruit extracts in animal models. However, little is known about their potential effects in humans, and very little is known about the mechanisms involved. This study aimed at identifying discriminant metabolites after acute and chronic intake of golden berry. Method: An untargeted metabolomics strategy using high-performance chemical isotope-labelling LC-MS was applied. The blood samples of eighteen healthy adults were analysed at baseline, at 6 h after the intake of 250 g of golden berry (acute intervention), and after 19 days of daily consumption of 150 g (medium-term intervention). Results: Forty-nine and 36 discriminant metabolites were identified with high confidence, respectively, after the acute and medium-term interventions. Taking into account up- and downregulated metabolites, three biological networks mainly involving insulin, epidermal growth factor receptor (EGFR), and the phosphatidylinositol 3-kinase pathway (PI3K/Akt/mTOR) were identified. Conclusions: The biological intracellular networks identified are highly interconnected with the insulin signalling pathway, showing that berry intake may be associated with insulin signalling, which could reduce some risk factors related to metabolic syndrome. Primary registry of WHO.

## 1. Introduction

Golden berry (*Physalis peruviana* L.) is a fruit of high commercial importance in some African and Latin American countries, where it is locally consumed and often exported to northern markets, mainly Europe and the US [1]. The traditional pharmacopoeia is relatively extensive concerning the potential health benefits of the berry, although it concerns mostly the use of calyces [2]. Nonetheless, folk medicine attributes antispasmodic, diuretic, antiseptic, sedative, and analgesic effects to the fruit [2]. When considering scientifically based studies, most reviews of the literature conclude that the strongest evidence indicates a hypoglycaemic effect and the improvement of insulin sensitivity [3]. Up to four independent studies conducted in India, Colombia, and Peru reported antidiabetic properties. Three interventions with golden berry extracts on streptozotocin-induced diabetic rats [4,5,6] and normal mice [7] showed positive effects on glycaemic and insulin related metabolites. The fourth study was conducted on 26 young human adults [8], and the authors reported that golden berry intake induced a postprandial decrease in glycaemia following a per os glucose challenge. Less documented is an additional potential positive impact of golden berry fruit consumption on oxidative stress and inflammatory processes and status. Nonetheless, most of these studies were conducted in vitro or using animal models, and data on human cohorts are very scarce. Additionally, very little is known about the mechanisms and compounds involved in the observed effects.

The fruit of *Physalis peruviana* L. contains a wide diversity of biochemical compounds. withanolides and their derivatives are the most emblematic metabolites of Physalis species, and they have been shown to exert a wide range of pharmacological activities in vitro, such as immunomodulatory, angiogenesis inhibitor, anticholinesterase, antioxidant, antibacterial, and antitumoral activities [9]. This family of compounds with a steroid backbone has attracted the attention of pharmacologists, as withanolides and derivatives are mostly concentrated in aerial parts of plants, such as the leaves. They were also detected in fruit pulp [10] but at low concentrations. Nonetheless, it is not clear whether such compounds could be bioaccessible and bioavailable for humans [11]. The fruit also contains an appreciable amount of lipids and carotenoids (essentially β-carotene [12]), with some lutein diesters [13], phytosterols [14], tocopherols [14,15], and flavonoids, essentially rutin [16]. Some of these compounds accumulate within diminutive seeds, which are probably not disrupted during passage through gastrointestinal tracts. However, in the case of golden berry, the levels of tocopherols and phytosterols are apparently much higher within the pulp and peel [14], which probably makes them more bioavailable. Therefore, compared with other fruit, golden berry is probably an important source of tocopherols (~17 mg/100 g of fruit FW, mainly γ, α, and β in order of importance) with high vitamin E activity and phytosterol content (~10 mg/100 g of fruit FW, mainly 5-avenasterol and campesterol) [14]. Additionally, it was recently shown that golden berry fruits could also be a source of trans-resveratrol, which is even richer than red wine [17]. On the other hand, specific disaccharide hydroxyesters have also been detected in golden berries [18,19]. These compounds have been associated with the inhibition of alpha-amylase, which could also contribute to the hypoglycaemic effect. Despite the high diversity of compounds present in golden berries, the potential health impacts after fruit consumption cannot yet be attributed to one specific molecule or group of compounds. This could potentially result from synergistic effects of many of these secondary metabolites.

The objective of this paper is to identify the changes in plasma metabolites that may occur after acute and medium-term ingestion of golden berry. To reach this goal, an untargeted metabolomic approach was used, but instead of relying on dubious identification and quantification of metabolites, chemical isotope labelling (CIL) coupled with liquid chromatography mass spectrometry was used. This chemical derivatization-based approach is revolutionizing untargeted metabolomics, as almost all metabolites with different polarities and functional groups can be analysed on the same reverse-phase column, following the same conditions under a positive ionization mode [20]. Unlike conventional LC-MS methods, it allows a high quantification accuracy, as CIL LC-MS uses differential isotope labelling to overcome the matrix effect, ion suppression effect, and instrument drift issue during sample analysis [21]. CIL LC-MS is particularly appropriate for profiling plasma metabolites and has been applied to detect discriminant metabolites in animal and human biofluids [22].

## 2. Materials and Methods

### 2.1. Plant Material

Fruits from the variety “Dorada” selected by AGROSAVIA (Ref. ICA UCH-16-02) were grown and collected by the company Caribbean Exotic S.A.S. at their farm “La Bendicion” (6.23779° latitude/−75.32.45° longitude) in San Vicente Ferrer, Antioquia, Colombia. The calyxes were manually removed, and selected fruits were packed in a plastic box and delivered to volunteers under refrigeration. All operations were carried out at the export packing warehouse following the strictest hygiene rules according to good agriculture practice (GAP) and good manufacturing practice (GMP).

### 2.2. Subjects

The sample size is 18, which is within the range used usually by high-throughput metabolomics studies for the identification of dietary discriminant metabolites [23,24]. Only males were used in this nutritional intervention as sex of the subjects known to strongly impacts metabolites profile such as amino acids, lipids, sugars, and keto acids [25].

The male volunteer subjects met the following inclusion criteria: aged 20–50 years old, BMI between 18.5 and 29.9 kg/mt^2^, regular consumers of citric fruits, non-smokers, physical activity of <10 h/w, no history and/or diagnosis of chronic disease (cardiovascular, gastric, or psychiatric disorder, dyslipidaemia, diabetes, cancer, renal or liver alteration, hypothyroidism, insomnia, dysautonomia, or sleep disturbance), not currently consuming medication (lipid-lowering drugs, antioxidant dietary supplements, anticonvulsants, anti-inflammatory steroids, or hypnotics) and not treated with antibiotics or anti-parasitic drugs for the last three months before the beginning of the study. The main clinical parameters of volunteers observed at the beginning of the study are presented in Supplementary Table S1. During the study, the consumption of food supplements was forbidden. The study trial was approved by the ethical committee of the Institute of Health Sciences of CES University (Medellin, Colombia; ethical ID: 707, 20 September 2017) and registered with the International Clinical Platform (https://rpcec.sld.cu/trials/RPCEC00000268-En; accessed on 17 April 2018). The study was conducted in strict accordance with the principles of the Declaration of Helsinki, as revised in 2013, the Nuremberg code, and the guideline of Council for International Organizations of Medical Sciences (CIOMS) 2016 and had minimal risk according to the Colombian Ministry of Health (Resolutions 008430/1993 and 2378/2008). Participants were assured of anonymity and confidentiality. All participants provided written informed consent.

The subjects were characterized in terms of some anthropometric and biochemical parameters to meet the inclusion criteria. Weight and height were measured by standardized specialized personnel using internationally accepted equipment and techniques. BMIs were calculated and classified according to WHO criteria. Blood pressure was obtained according to the standard techniques of the AHA and the European Cardiology Association. The subjects rested between 3 and 5 min. The cut-off for normal blood pressure was 120/80 mm Hg. Plasma TC, HDL cholesterol, and TGs were enzymatically measured with cholesterol oxidase/peroxidase, cholesterol HDL direct, and TG glycerol phosphate oxidase/peroxidase commercial kits (BioSystems S.A). The LDL was calculated. The cut-offs were according to The National Library of Medicine, National Health Institute (NIH) of the USA. Hepatic transaminases (AST and ALT) were determined by spectrophotometry, and reference values were those proposed by the NIH for men. Blood creatinine was measured by the colorimetric method, and a normal result for men was obtained according to the NIH (0.7 to 1.3 mg/dL). Fasting glucose was evaluated by an ultraviolet irradiation assay, and normal values were 70–100 mg/dL, fasting insulin was evaluated by a chemiluminescence immunoassay, and values in the range 2.6–24.9 μUI/mL were considered normal. Glycated haemoglobin was determined by high-performance liquid chromatography, and values less than 5.7% were considered normal. Blood glucose and insulin were used to calculate the insulin resistance index using the homeostasis model assessment (HOMA-IR) and the cut-off to normality better reflected the biochemical characteristics of the studied population (HOMA-IR > 3).

### 2.3. Study Design

This pilot study lasted 3 weeks. The study had three time points: the washout (one week), baseline, and intervention periods (19 days). During the washout period, the volunteers were asked to avoid consumption of any type of golden berry. Blood samples were drawn by puncturing the antecubital vein to obtain the plasma as follows. One fasting sample and another blood sample 6 h after ingestion of 250 g of golden berry, breakfast, and lunch from a standard menu were withdrawn. Then, volunteers consumed 150 g of golden berry daily for 19 days, and at the end of the intervention, a fasting blood sample was withdrawn 24 h after the last ingestion of golden berry fruits. On the days of sampling, the same standardized meals were provided to volunteers. The plasma was preserved at −80 °C until analysis.

### 2.4. Phytochemical Analysis of Fruits

A sample of fruits used in the nutritional intervention was separated for analysis. Moisture, protein, fat, total carbohydrate, fibre, ash, and energy were determined according to AOAC standard methods. Total phenolic were performed after extraction with acetone/water (70/30) following the classical Folin-Ciocalteu method modified by [26] and expressed in equivalent gallic acid. Carotenoids were analysed by an Agilent 1100HPLC-DAD system (Massy, France). Carotenoids were extracted and separated using a C30 column (150 mm × 4.6 mm i.d., 3 µm) (YMC EUROP GmbH, Dinslaken, Germany) as detailed recently by [27]. Total carotenoids was expressed in equivalent All-E-β-carotene and pro-vitamin A as mcg retinol activity equivalent (RAE) taking a bioconversion ratio of 12:1 for all-E-β-carotene. Finally, vitamin C was assessed by HPLC according to The United States Pharmacopeial Convention (USP 36).

### 2.5. Plasma Sample Preparation

Plasma samples were thawed, vortexed, and centrifuged at 15,000× *g* for 15 min at 4 °C. An aliquot of 250 μL of the supernatant was mixed with precooled LC-MS-grade methanol (3:1 *v*/*v*) and vortexed thoroughly to allow protein precipitation. The mixture was left at −20 °C for one hour, vortexed and spun down for 20 min, and centrifuged again as described above. The recovered supernatant (750 μL) was completely dried using a centrifugal vacuum concentrator (Labconco, Kansas City, MO, USA). Each individual’s plasma sample was centrifuged and, then, redissolved in 250 μL of LC-MS grade water. After vortexing and centrifugation, the supernatant was collected and split into 6 aliquots for different labelling methods, backup, and preparation of the pooled sample. An aliquot of 50 μL was taken from each individual sample and, then, combined and mixed thoroughly to prepare the pooled sample, which was used as a quality control (QC). Data were analysed using a Dansyl-Labelling Kit for Amine and Phenol Metabolomics I (Nova Medical Testing Inc., Edmonton, AB, Canada). The labelling protocol followed was described in detail by Zhao et al. [21]. For amine/phenol aliquot labelling, the labelling protocol strictly followed the SOP provided in the kit. Briefly, 12.5 μL of buffer reagent and 37.5 μL of ^12^C_2_-labelling (for the individual samples and the pooled sample) or ^13^C_2_-labelling (for the pooled sample) reagent were added to the samples. After being vortexed, the mixtures were incubated at 40 °C for 45 min. Then, 7.5 μL of quenching reagent was added to neutralize the excess of labelling reagent followed by an incubation at 40 °C for another 10 min. Finally, pH was adjusted with 30 μL of dedicated reagent.

Quantification of labelled metabolites was performed by LC-UV after centrifugation at 15,000× *g* for 10 min. The result was used for preacquisition normalization for all four-channel metabolomics analyses. The ^12^C_2_-labelled individual sample was mixed with a ^13^C_2_-labelled reference sample in equal amounts according to LC-UV normalization results. The entire sample set and quality control (QC) sample were prepared by an equal-amount mix of ^12^C-labelled and ^13^C-labelled pooled samples.

### 2.6. Analysis by LC-MS

Mixed samples were analysed by LC-MS (Agilent 1290 LC linked to Bruker Impact II QToF mass spectrometer) according to the conditions described previously [20]. Briefly, an Agilent eclipse plus reversed-phase C18 column (150 × 2.1 mm, 1.8 μm particle size) was used with mobile phases A (0.1% (*v*/*v*) formic acid in water) and B (0.1% (*v*/*v*) formic acid in acetonitrile) following a gradient of t = 0 min, 25% B; t = 10 min, 99% B; t = 13 min, 99% B; t = 13.1 min, 25% B; and t = 16 min, 25% B at a flow rate of 400 µL/min and a column oven temperature of 40 °C. QC samples were injected every 20 sample runs to monitor the instrument performance. Calibration data were used to check the retention time. All calibration peaks were well aligned. Their retention times were consistent for all calibration data, showing good RT stability for data acquisition.

### 2.7. Data Processing and Cleansing

LC-MS data for the plasma samples were exported to a “.csv” file using Bruker DataAnalysis 4.4, and the data quality was checked. For all analyses, ions or peak pairs that were not present in at least 80% of samples in any group (baseline, acute and medium-term groups) were filtered out to ensure data quality. All data were normalized by the ratio of total useful signals.

### 2.8. Statistical Analysis

The datasets obtained were pre-treated using Pareto scaling and log transformation. Univariate and multivariate statistical approaches were performed using SIMCA-P software (Sartorius Stedim Data Analytics AB, Umea, Sweden) to explore the differences between sample groups. A principal component analysis (PCA) was performed with all detected ions to explore group differences. Afterward, a partial least-squares discriminant analysis (PLS-DA) using SIMCA P was applied to assess the variation between groups. The predictive ability of the model was validated by cross-validation ANOVA (CV-ANOVA) and permutation tests (*n* = 100) to test the eventual overfitting of the data. The variable’s importance in the projection (VIP) of the PLS-DA model, which summarizes the contribution of the variable to the model, was registered. The VIP score of a variable is calculated as a weighted sum of the squared correlations between the PLS-DA components and the original variable. The weights correspond to the percentage of variation explained by the PLS-DA latent component in the model. All ions with a *p*-value < 0.05 (from univariate analysis) and a VIP > 1 (variable importance (VIP) of partial least-squares multivariate discriminant analysis) were considered statistically significant and discriminant between groups. The database for statistical analyses is available at Github (https://github.com/Vidarium/Biomarkers-of-exposition_Golden-berry, accessed on 2 September 2021).

### 2.9. Metabolite Identification

For the identification of metabolites, a three-tier ID approach was used. In tier 1, peak pairs were matched against an in-house labelled standards metabolite library (CIL Library) based on triple parameters, accurate molecular masses, retention times (RT), and MSM/MS spectra. The parameters of mass and retention time tolerance for this CIL library were fixed at 10 ppm and 30 s, respectively. In tier 2, a linked identity library (LI Library) was used for identification of the remaining peak pairs. The LI Library includes over 7000 pathway-related metabolites, providing high-confidence putative identification results based on accurate masses and predicted retention times. For this LI library, the parameters of mass and retention time tolerance were fixed at 10 ppm and 205 s, respectively. Metabolites with no match in tier 1 or 2 were reported as “Unknown”. In some cases, although the identification of the enantiomers was not possible, the putative attribution of the L form was made taking into account the logical biological interpretation based on the literature, as for example, when only the L form has been reported in the blood.

### 2.10. Discovery of Biological Association Networks

For the discovery of association networks, Ingenuity Pathway Analysis (IPA) software (Ingenuity, Redwood City, CA, USA) was used. The KEGG IDs and log2 FC (fold-change) of identified metabolites were input to seek potential interactions with other biological molecules. The *p*-values were calculated using Fischer’s exact test to determine the probability that the association between the metabolites in the dataset and the top pathway was due to chance alone. IPA uses a network-generation algorithm to segment the network map between metabolites into multiple networks and assign scores for each network [28]. The score is generated based on a hypergeometric distribution, where the negative logarithm of the significance level is obtained by Fisher’s exact test at the right tail. For canonical pathway analysis, disease, and function, a − log (*p*-value) > 2 was taken as the threshold, a Z-score > 2 was defined as the threshold of significant activation, and a Z-score < −2 was defined as the threshold of significant inhibition. For regulator effects and molecular networks, consistency scores were calculated, in which a high consistency score indicated accurate results for the regulatory effects analysis. For upstream regulators, the *p*-value of overlap <0.05 was set as the threshold. The algorithm used for calculating the Z-scores and *p*-values of overlap has been described previously [29]. For multiple comparisons, *p*-values were adjusted using Bonferroni correction. The biological interpretation was accomplished using available information from the literature, as well as web-based software tools, such as BioCyc [30], KEGG [31], and ChEBI [32].

## 3. Results

The main phytochemical composition of golden berry fruits used in the nutritional intervention is presented in Table 1. The fruit was analysed without calyx and seeds because the small lignified seeds are neither chewed nor digested. Data show an important content in total carotenoids and pro-vitamin A, vitamin C, and total phenolic compounds.

**Figure 1:**
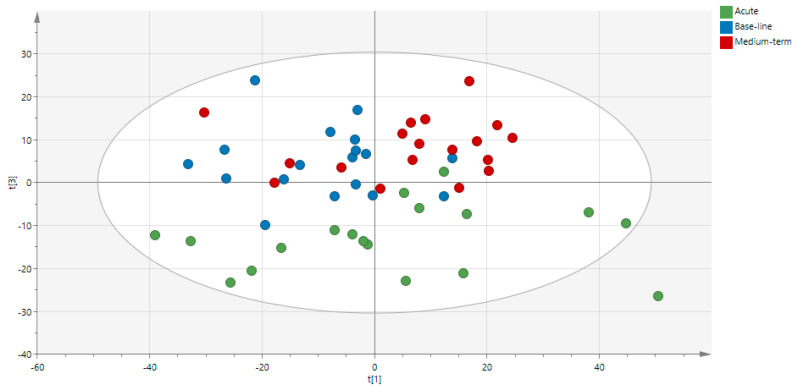
Non-supervised PCA of the plasmatic metabolome at baseline (before consumption of *Physalis peruviana* L.) and after acute consumption and medium-term consumption.

After injection of plasma samples collected during nutritional intervention with golden berries, an average of 1953 peak pairs per run was detected. The unsupervised PCA for the plasma metabolome showed differences—in the space constituted by PCA 1 and 3 (Figure 1)—between the baseline, acute, and medium-term consumption levels. Along the PC1 axis, which recovers approximately 19% of the explained variance, a global separation between baseline and medium-term consumption can be observed for most individuals. Along the PC3 axis, which recovers approximately 7% of the information, the plasma metabolome after acute consumption appears in a different space than that at baseline. Nonetheless, it can be observed that after medium-term consumption, a minority of individuals have a metabolome similar to that at baseline. Notably, plasma from medium-term exposure subjects was collected 24 h after the last ingestion of golden berries, and some individuals had a plasma metabolome similar to that at baseline.

**Figure 2:**
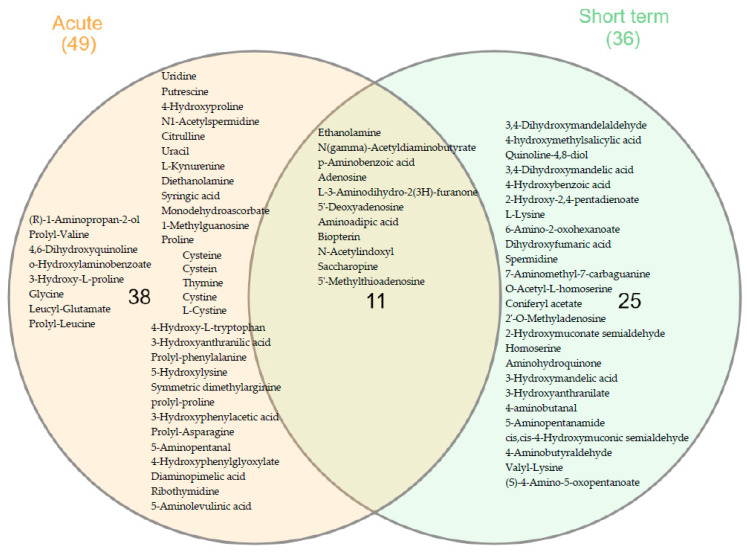
Venn diagram of the main discriminant metabolites identified after acute and medium-term intervention (only metabolites with VIP > 1.2 and *p*-value < 0.05 are shown).

The supervised PLS-DA models for baseline vs. acute and baseline vs. medium-term show good statistical performances with very low probability of overfitting the data for both models (see Figures S2 and S3: for baseline vs. acute: R2Y = 0.993, Q2Y = 0.895, *p*-value CV-ANOVA = 2.03 × 10^−9^, intercept cross-validation = −0.14, and for baseline vs. medium-term: R2Y = 0.975, Q2Y = 0.7, *p*-value CV-ANOVA = 3.75 × 10^−6^, intercept cross-validation = −0.08). On the basis of the PLS-DA (VIP > 1.2) and *p*-values (<0.05), a list of discriminant metabolites between groups was established. Among them, 49 metabolites for acute intervention and 36 after medium-term intervention could be putatively identified with a relatively high confidence. As reported in the Venn graph (Figure 2), 11 metabolites were shared between acute and medium-term metabolomes. In total, only 74 compounds were putatively identified, with 11 of them being isomers with the same mass as the original compounds but different retention time. The complete table is provided in the Supplemental Information (Tables S2 and S3).

### 3.1. Identification of Biological Association Networks

#### 3.1.1. Baseline and Acute Nutritional Intervention with Golden Berries

**Figure 3:**
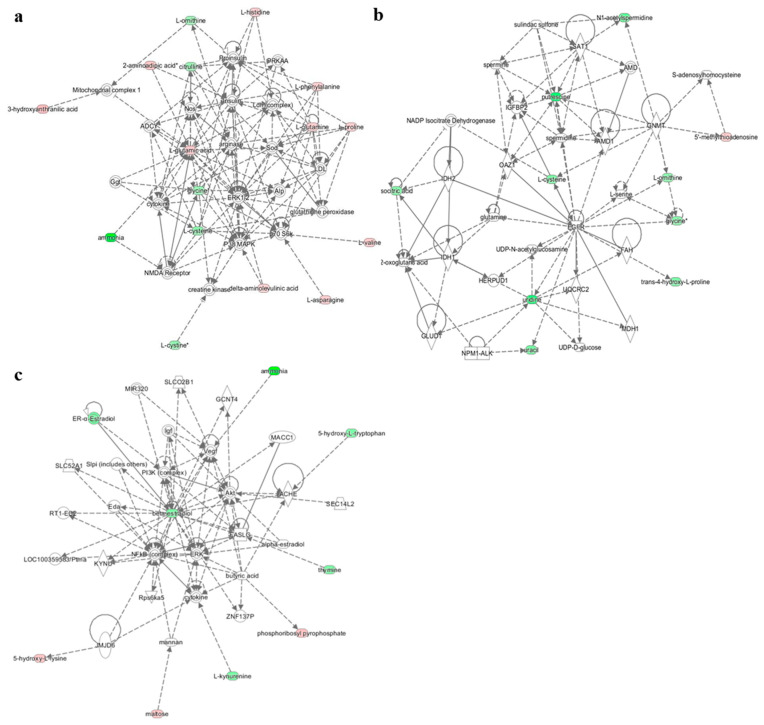
Significant biological networks associated with acute intervention with golden berry fruits. (**a**) Network associated with developmental disorders, hereditary disorders, and metabolic diseases. (**b**) Network associated with small-molecule biochemistry, molecular transport, and amino acid metabolism. (**c**) Network associated with cell morphology, cellular assembly and organization, and cellular development. Metabolites are represented as nodes. The biological relationship between two nodes is represented as a line. Note that the coloured symbols represent metabolites that occur not only in our data but also in Ingenuity^®^ pathways correlations, while the transparent entries are molecules from the Ingenuity Knowledge Database. Metabolites highlighted in red have increased levels, whereas metabolites highlighted in green have decreased levels. Solid lines between molecules indicate a direct physical relationship between the molecules, and dotted lines indicate indirect functional relationships.

Among the 55 identified metabolites that were significantly changed after the acute intervention, 27 were downregulated, and 28 were upregulated (Supplementary Table S2). To identify possible biological associations between these metabolites, bioinformatic computation analysis was performed (IPA software, the KEGG IDs, and log2 FC (fold-change)) on the 60 previously identified metabolites. As shown in Figure 3, most of the discriminant metabolites were significantly connected (top score) in three main networks: (1) developmental disorders, hereditary disorders, and metabolic diseases (Figure 3a), (2) small-molecule biochemistry, molecular transport, and amino acid metabolism (Figure 3b), and (3) cell morphology, cellular assembly as well as organization, and cellular development (Figure 3c). Although all participants were healthy subjects, the analysis of the network “Developmental disorders, hereditary disorders and metabolic diseases” showed that insulin and insulin signalling metabolites have key roles in the integration of the discriminant metabolites after acute ingestion of golden berries and the intake of a standardized breakfast and lunch (postprandial). Within the network “Small-molecule biochemistry, molecular transport and amino acid metabolism” (Figure 3b), the discriminant metabolites associated with the epidermal growth factor receptor (EGFR) were all downregulated in the same period. Finally, the analysis of the network “Cell morphology, cellular assembly and organization and cellular development” (Figure 3c) showed the integration of some metabolites within the phosphatidylinositol 3-kinase pathway (PI3K/Akt/mTOR).

Considering up- and downregulated discriminant metabolites, IPA software predicted significant effects (*p* < 0.05) related to some cellular functions and physiological system development. Regarding cellular functions, the top associated categories were protein synthesis (*p*-value = 1.59 × 10^−7^) (Supplementary Figure S1a), cellular growth and proliferation (*p*-value = 2.17 × 10^−6^) (Supplementary Figure S1b), and amino acid metabolism and molecular transport (*p*-value = 1.09 × 10^−5^) (Supplementary Figure S1c). Meanwhile, for physiological system development and function, the most relevant effect was related to hepatic system development and function (*p*-value = 7.78 × 10^−8^), suggesting possible participation in the maintenance of normal liver function (Supplementary Figure S1d) and immune cell trafficking (*p*-value = 8.35 × 10^−7^), with a role in the recruitment and performance of immune system cells (Supplementary Figure S1e). Interestingly, some metabolite networks were activated, including alkaline phosphatase (Supplementary Figure S1f), albumin (Supplementary Figure S1g), potassium levels (Supplementary Figure S1h), and lactate dehydrogenase (LDH) release (Supplementary Figure S1i).

#### 3.1.2. Baseline and Medium-Term Intervention with Golden Berry

**Figure 4:**
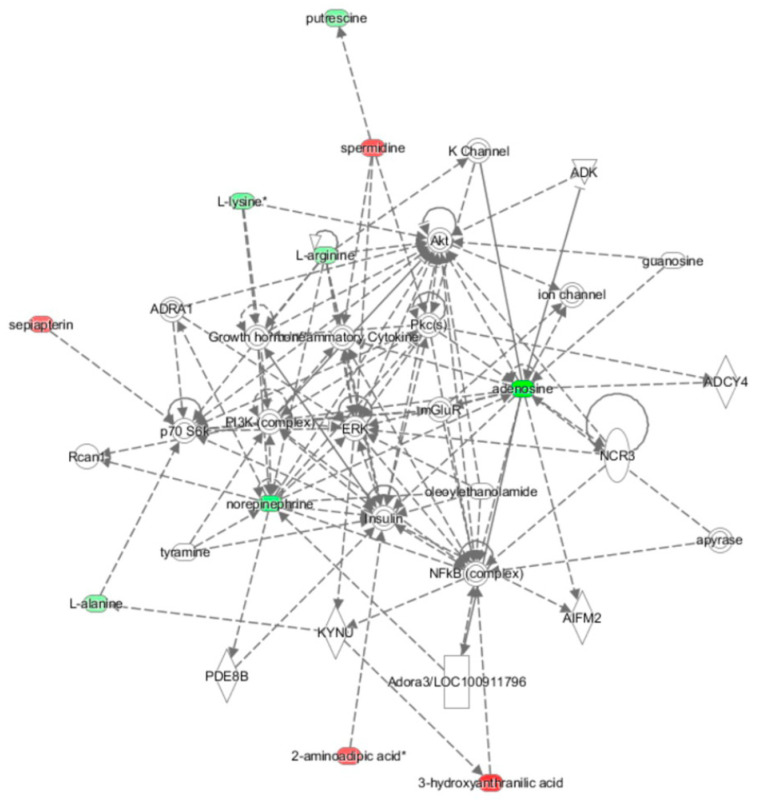
Biological networks “DNA replication, recombination, repair, molecular transport and nucleic acid metabolism” in which significant associations were found between previously identified discriminant metabolites (*p* < 0.05) after medium-term intervention with golden berry fruits.

The metabolomic analysis enabled us to tentatively identify 41 metabolites in plasma with high confidence after consuming golden berry fruits—14 were downregulated, and 27 were upregulated (Supplementary Table S3). The IPA metabolomic analysis between up- and downregulated metabolites showed integration within one top network associated with DNA replication, recombination, and repair, molecular transport, and nucleic acid metabolism. As shown in Figure 4, the discriminant metabolites could be associated with insulin and the phosphatidylinositol 3-kinase pathway (PI3K/Akt/mTOR).

IPA software predicted significant effects on molecular and cellular function, especially cell-to-cell signalling (*p*-value = 1.89 × 10^−8^), cellular movement (*p*-value = 3.06 × 10^−6^), cell signalling (*p*-value = 1.04 × 10^−5^), small-molecule biochemistry (*p*-value = 4.20 × 10^−4^), and protein synthesis (*p*-value = 4.21 × 10^−3^). Regarding physiological system development and functions, the software predicted significant effects on cardiovascular system development (*p*-value = 5.12 × 10^−6^), haematological development (*p*-value = 4.08 × 10^−7^), organ morphology (*p*-value = 3.05 × 10^−9^), organismal development (*p*-value = 3.06 × 10^−5^), and immune cell trafficking (*p*-value = 1.56 × 10^−8^).

## 4. Discussion

The analysis of all the biological networks significantly mobilized after acute and medium-term consumption of golden berry showed strong associations with insulin and insulin signalling due to the integration of most discriminant metabolites. Proinsulin, insulin, and insulin signalling are directly at the centre of the first network built by IPA software around discriminant metabolites (Figure 3a). For the other two networks that were significantly changed, discriminant metabolites pointed to EGFR (Figure 3b) and PI3K/Akt/mTOR (Figure 3c). After medium-term consumption of golden berries, only the PI3K/Akt/mTOR pathway remained altered (Figure 4). All these pathways are highly interconnected with insulin signalling.

The insulin signalling pathway plays two major roles in cells, both metabolic and mitogenic. First, it regulates metabolic processes such as carbohydrate, lipid, and protein metabolism. Second, it modulates cell division and growth through its mitogenic effects [33].

In the case of the acute response to golden berry consumption, the effect on the insulin signalling pathway could be due either to the fruit or to the meal that was ingested. Nonetheless, insulin was also at the centre of the medium-term biological network (Figure 4), which compared two fasting plasma metabolomes. Thus, although there may have been interference during the acute intervention, our results seem to reinforce the results already observed in vivo in animal models [5,34]. In mammals, insulin plays a key ubiquitous role in energy homeostasis. It influences the expression and activity of a variety of channels and enzymes involved in the metabolic processes of glucose transport, glycogenesis, glycogenolysis, glycolysis, and inhibition of gluconeogenesis in the liver [33,35,36,37]. Disruption of these mechanisms induces insulin resistance. This poor insulin signalling is a foundational aspect of the pathogenesis of metabolic syndrome, obesity, type 2 diabetes, and most chronic diseases and comorbidities linked with an unhealthy diet, e.g., cardiovascular diseases and cancer [30,31,33].

At the molecular level, insulin actions on insulin-sensitive tissues such as liver, muscle, and adipocytes are mediated by its membrane receptors. Activation of insulin receptor (IR) tyrosine kinase reflects insulinaemia and is associated with decreased glycaemia. Upon insulin binding to IR, the receptor is autophosphorylated to subsequently trigger intracellular signalling pathways. These pathways are organized into a complex network of protein interactions and phosphorylation cascades at both the cytosolic and nuclear levels. Two main pathways are mobilized: (1) the PI3K/Akt/mTOR pathway through pleiotropic IRS docking molecules and (2) the Shc/GRB2/SOS/Ras/MAPK pathway. Both pathways control most insulin metabolic actions, such as energy metabolism (carbohydrates, lipids) and gene expression (cell proliferation, differentiation, and growth) [38,39]. These cellular effects correspond to the dual potential of insulin, which behaves as both a hypoglycaemic hormone and an anabolic growth factor. The PI3K pathway also has a key role in the integration of up- and downregulated metabolites for the activation of the insulin signalling pathway [39], confirming the significant effects on cell-to-cell signalling, cell movement, cell signalling, and protein synthesis predicted by IPA software.

A preclinical study already suggested that the consumption of golden berry juice decreased blood glucose and insulin resistance and increased insulin levels in diabetic rats [40]. Nevertheless, the glucose detected in plasma did not appear as a discriminating metabolite, so we can assume that glucose level was not significantly affected. However, we report for the first time that the relationship in the insulin signalling pathway is supported by integration into the relevant networks of plasma metabolites that are significantly modified after acute ingestion of golden berry fruit. The effect tends to fade after discontinuing consumption of the fruits. Even after medium-term fruit consumption, the effect declined 24 h after the last ingestion (Day 19), remaining significantly altered only at the level of PI3K/Akt/mTOR.

Following golden berry ingestion, predictions also mentioned relations with the immune system through modulation of cytokine levels, inflammation, and the NFKB signal transduction channel. Low-grade inflammatory processes are common to diseases associated with energy metabolism (metabolic syndrome, diabetes, obesity, etc.), as well as with tumorigenesis [41]. Our results suggest a modulatory influence of *Physalis peruviana* ingestion. Inflammation in metabolic diseases is also detrimental to the cardiovascular system. It is, therefore, interesting to note that the pathway analysis revealed an association with arginase. This enzyme was demonstrated to modulate NO levels in vascular endothelial cells and smooth vascular muscles, thereby impacting arterial blood pressure regulation [42]. Additionally, our metabolomic results identified norepinephrine variations. This endogenous neurotransmitter from the sympathetic autonomic nervous system is involved in both energy expenditure and blood pressure control through its actions on beta-1 cardiac receptors and alpha-1 vascular receptors [43]. These results could indicate a potential combination of effects with NO pathways on the cardiovascular system in relation to glucose and lipid metabolism.

Research on tyrosine kinase receptors, such as EGFR, revealed the mechanisms of their activation by growth factor ligands [44]. The EGFR tyrosine kinase intracellular network main channel is the Shc/GRB2/SOS/Ras/MAPK pathway. This signalling cascade controls cell proliferation and differentiation processes. In addition, EGFR is also able to mobilize the PI3K/Akt/mTOR, p53, Ras/MAPK, and NFKB pathways to regulate cell proliferation/growth, amino acid metabolism, cell survival/apoptosis, and cell morphology/motility. In addition, EGFR is known to induce the nuclear factor NFKB via PIK/Akt and MAPK signalling to regulate the immune system and associated inflammation processes, as well as angiogenesis [45]. In this context, our results showed a significant impact of acute ingestion of golden berry fruit on EGFR. Almost all discriminant metabolites involved within its biological network were significantly changed (Figure 3c). These results showed that acute consumption of golden berry may affect the activity of EGFR tyrosine kinase. Furthermore, numerous studies have shown the potential contributions of EGFR-associated signalling pathways in oncogenesis processes, including cell proliferation, angiogenesis, and resistance to apoptosis [46]. In this context, our results suggest that golden berry fruit consumption as part of a healthy diet could negatively influence EGFR signalling. These combined observations are in accordance with previous reports indicating antitumoral effects observed in a rat model after ingestion of golden berry juice [47]. Taken together, these data suggest the anti-oncogenic potential of *Physalis peruviana*. Based on our results, the consumption of the fruits appeared to influence EGFR, which was reported to be involved in a diversity of tumours. These effects might be explained by a loss of EGFR binding affinity, since this receptor is regulated by protein kinase C [44,48]. Alternatively, other more complex mechanisms yet to be discovered could involve modulation of EGFR intracellular signals through crosstalk with insulin signalling. One element that may support this hypothesis is in the link that our results identified between EGFR signalling and insulin-growth-factor binding protein 2 (IGFBP2). This endogenous compound is currently considered a major discriminant metabolites but also regulator of insulin resistance and associated metabolic processes in relation to insulin signalling cascades [48].

A possible explanation for the results we obtained may lie in the golden berry fruit composition and relative contents of various bioactive compounds. One of these compounds, which is not specific to this fruit, is β-carotene [12]. This bioactive molecule can lead to the activation of PI3K/Akt/mTOR [34] insulin receptor substrate 1 (through IRS-1) phosphorylation, promoting insulin signalling pathway activation and thereby reducing insulin resistance [49]. In this context, evidence also suggests that a diet enriched in carotenoids with pro-vitamin A functions, such as β-carotene, improves liver function, which is insulin-sensitive tissue [47,50]. The other type of molecule, withanolides, is present in the Physalis sp. composition. Among these compounds, withangulatin-A has previously demonstrated insulin-release stimulatory effects in induced diabetic rats, similar to the reference drug glibenclamide (a potassium channel blocker on endocrine Langerhans insulin-secreting cells), suggesting antidiabetic potential through modulation of insulin levels and glucose homeostasis [51]. Relatedly, our metabolome analysis also indicated potential impact on potassium levels (Supplemental Figure S1h) after golden berry fruit consumption, but potassium levels in plasma were not measured. Other compounds are much more specific to *Physalis peruviana*, such as peruviosides, which are sucrose esters that exhibit important inhibition of α-amylase [18]. These compounds could also contribute to hypoglycaemic activity, thereby also affecting insulinaemia. These sugars were not detected in the plasma. Nonetheless, a synergistic effect between carotenoids, withanolides, and sucrose esters, along with other unknown compounds, should not be ruled out and could explain our observations. Our findings and mechanistic hypothesis based on our metabolomic approach are supported by a recent report from Pino-de-la Fuente et al. Indeed, the authors recorded positive effects of *Physalis peruviana* in vivo, i.e., insulin resistance and inflammation improvement in the muscles and liver in a mouse model of diet-induced obesity. These data point in the same direction as ours at the molecular and physiological levels in relation to the compositions of golden berry fruit, as suggested by the authors [52].

Taking into account up- and downregulated metabolites, as well as data from the literature, three biological networks could be integrated, involving mainly insulin, EGFR, and PI3K/Akt/mTOR. The PI3K pathway is common to insulin and EGFR signalling. These two growth factor molecules also share the MAPK signalling pathway. These signalling cascades control the metabolic and mitogenic effects of insulin and EGFR. Both biological intracellular networks are highly interconnected with the insulin signalling pathway. The insulin network was previously demonstrated to interact with other growth factor signalling pathways, such as EGF/EGFR, through bidirectional crosstalk. More specifically, the EGFR intracellular network, the Shc/GRB2/SOS/Ras/MAPK pathway, was reported to be linked to the insulin signalling cascade, with insulin regulating early signalling events of EGFR [38]. Therefore, our results suggest that different compounds from *Physalis peruviana* could be considered good candidates for use as beneficial modulators of pathophysiological processes involving insulin-associated cell dysregulation. Golden berry fruits seem to mobilize the signalling pathways common to the two endogenous anabolic molecules, namely, insulin and EGFR, leading to their direct or indirect modulation of energy and cell cycle homeostasis. These impacts and the technology used in our investigation point out the complex interplay between metabolic and mitogenic processes, wherein cell signalling controls metabolism and reciprocally controls metabolism signalling [53]. This integrative modulation of cell homeostasis appears to be partially oriented by the molecular contents of *Physalis Peruviana*, providing some insight explaining the beneficial effects of this fruit on health.

## 5. Conclusions

To our knowledge, the present study is the first investigation reported in the international literature regarding the effects of golden berries (*Physalis peruviana* L.) on insulin-pathway and anti-diabetic potential in a human model. The intake of golden berry fruit seems to be associated with insulin-related biological networks, as well as the EGFR and PI3K/Akt/mTOR pathways. Owing to the methodology followed, including untargeted metabolomics coupled with high-performance chemical isotope-labelling LC-MS, we were able to identify, with high confidence, plasmatic metabolites involved in these biological networks. This chemical derivatization-based approach was applied to an intervention with *Physalis peruviana* associated with the discovery of association networks using IPA software. This method allows a theoretical deduction in the impact on the human health of an intervention with *Physalis peruviana*. Following the method developed, it was shown that the ingestion of golden berries impacts insulin-associated signalling pathways, allowing us to propose hypotheses related to the potential mechanism of action through which *Physalis peruviana* could exert the biological activities already observed in preclinical studies. Currently, further clinical studies are required to evaluate the functional effects of golden berry consumption on insulin and glucose metabolism in healthy volunteers and volunteers with prediabetic and diabetic conditions.

## Figures and Tables

**Table 1:** Main nutrients and phytochemicals composition of golden berry fruits.

Compounds	Values
Moisture (g/100 g FW)	80–84
Protein (g/100 g FW)	0.03–1.6
Fat (g/100 g FW)	0.2–0.6
Carbohydrate (g/100 g FW)	16.2–19.6
Total sugar	8.5–10.5
Dietary fibres (g/100 g FW))	5–7
Ash (g/100 g FW)	0.79
Energy (Kcal/100 g FW)	53–80
Total phenols (gallic acid equivalents mg/100 g FW)	40–82
Beta-carotene (mcg/100 g FW)	1500–1917
Pro-vitamin A (mcg RAE/100 g FW)	95–125
Ascorbic acid (mg/100 g FW)	14–43

FW: fresh fruit pulp (without seeds and calyx); RAE: retinoic acid equivalents.

## Data Availability

The database for statistical analyses is available at Github (https://github.com/Vidarium/Biomarkers-of-exposition_Golden-berry) (accessed on 2 September 2021).

## Supplementary Materials

The following are available online at https://www.mdpi.com/article/10.3390/nu13093125/s1, Table S1: Baseline clinical characteristics of the volunteers, Figure S1: Metabolites regulatory networks associated with several function categories after acute nutritional intervention with *Physalis Peruviana*. (a) Regulatory network of protein synthesis. (b) Cellular growth and proliferation. (c) Molecular transport. (d) Improvement of the hepatic cell system. (e) Improvement of immune system cells. (f) Increase albumin. (g) Increase alkaline phosphatase. (h) Increase in potassium levels (i) and lactate dehydrogenase release, Table S2: List of discriminant metabolites identified with high confidence, significantly changed after acute nutritional intervention with *Physalis Peruviana* (compounds written normally tier 1 and in italics tier 2, Table S3: List of discriminant metabolites identified with high confidence, significantly changed after short-term nutritional intervention with *Physalis Peruviana* (compounds written normally tier 1 and in italics tier 2), Figure S2: Results of PLS-DA model “base-line Versus Acute” and permutation tests for its validation, Figure S3: Results of PLS-DA model “base-line Versus Short Term” and permutation tests for its validation.
