# Supplementary material for: Plasma Metabolome Profiling by High-Performance Chemical Isotope-Labelling LC-MS after Acute and Medium-Term Intervention with Golden Berry Fruit (Physalis peruviana L.), Confirming Its Impact on Insulin-Associated Signaling Pathways
Source: Nutrients. 2021 Sep 7;13(9):3125. doi: 10.3390/nu13093125 (PMC8468427; doi:10.3390/nu13093125)
Supplement: Supplementary file 1 [file nutrients-13-03125-s001.zip › nutrients-1314110-supplementary.pdf]

SUPPLEMENTAL MATERIAL

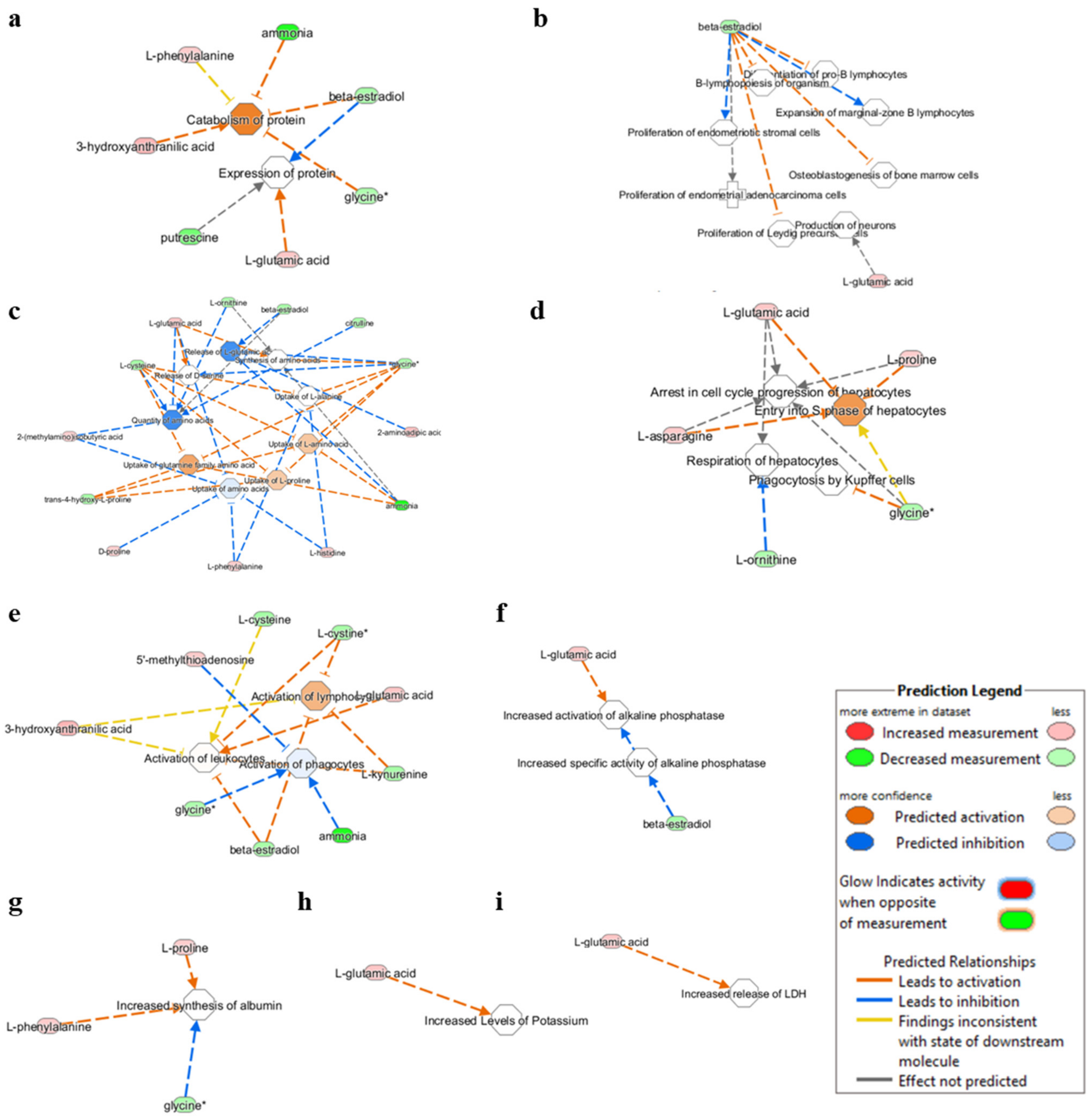

Figure S1: Metabolites regulatory networks associated with several function categories after acute nutritional intervention with Physalis Peruviana. **(a)** Regulatory network of protein synthesis. **(b)** Cellular growth and proliferation. **(c)** Molecular transport. **(d)** Improvement of the hepatic cell system. **(e)** Improvement of immune system cells. **(f)** Increase albumin. **(g)** Increase alkaline phosphatase. **(h)** Increase in potassium levels **(i)** and lactate dehydrogenase release.

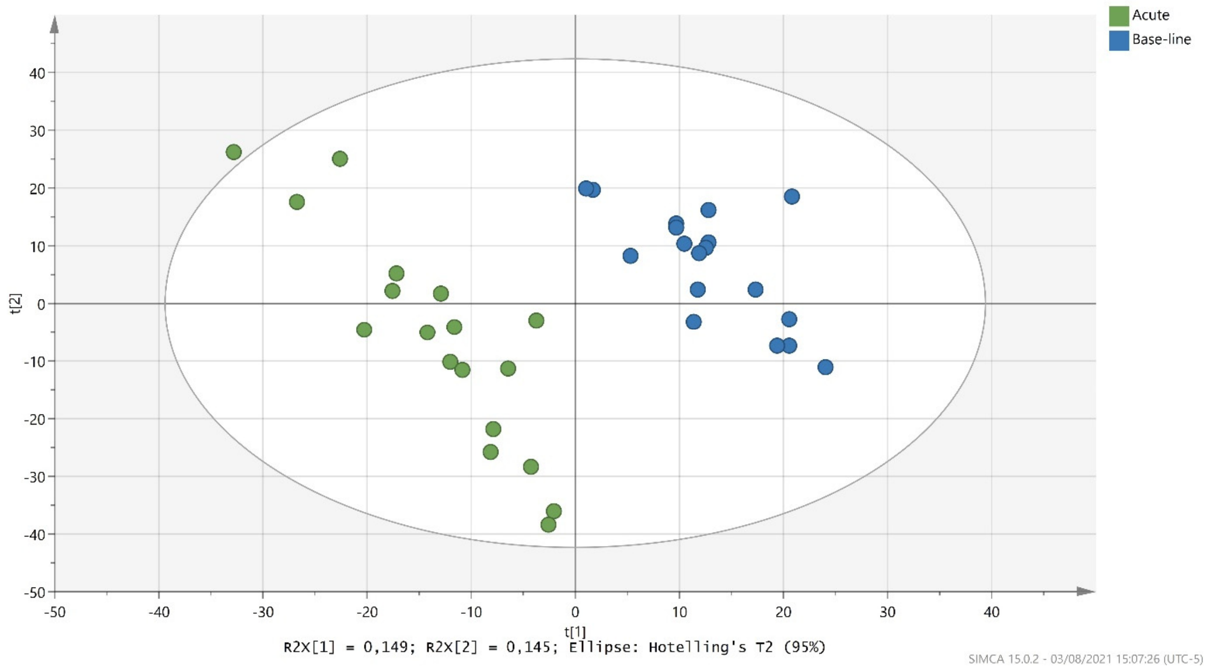

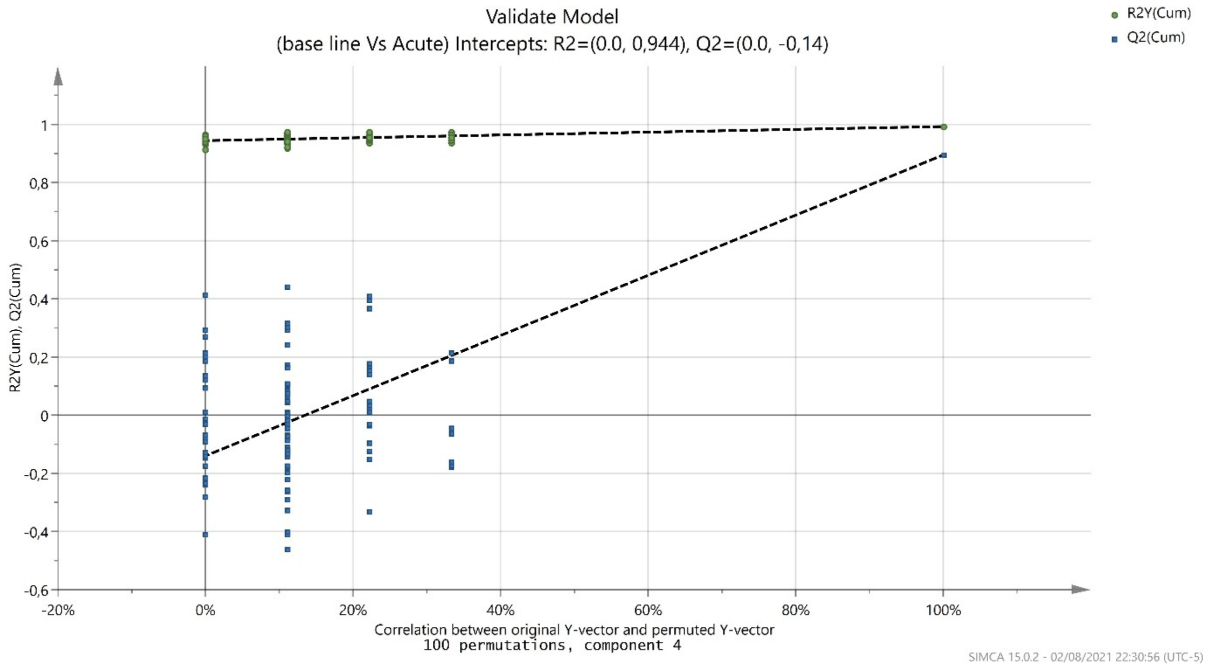

Figure S2: Results of PLS-DA model "base-line Versus Acute" and permutation tests for its validation

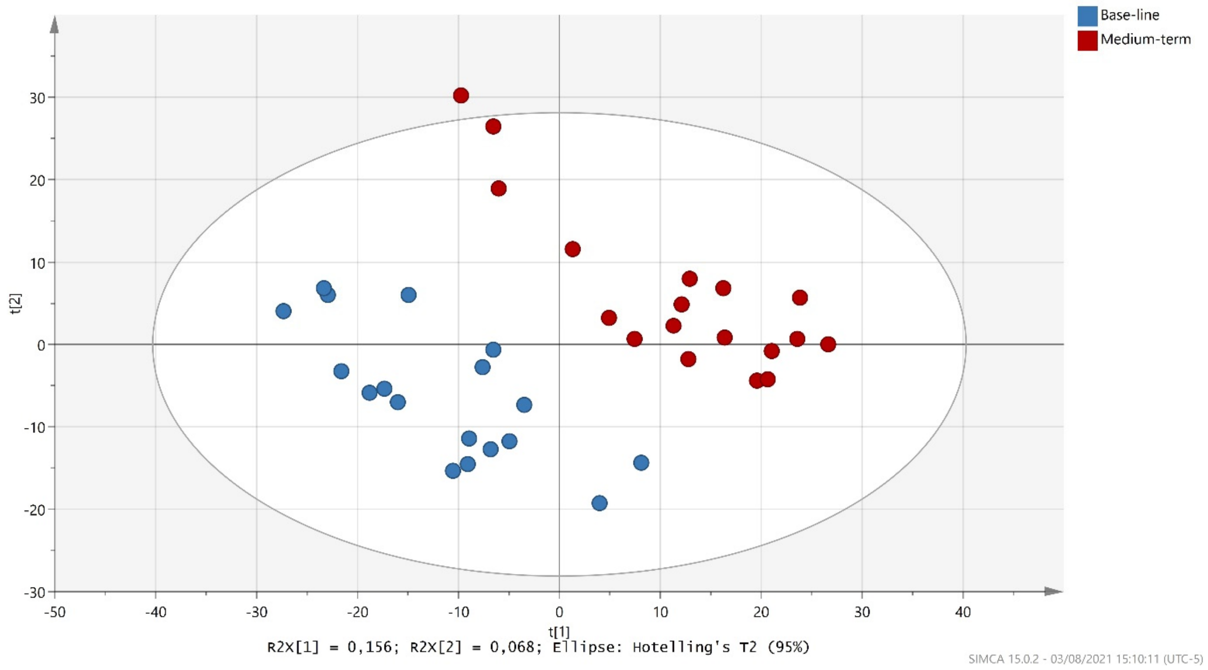

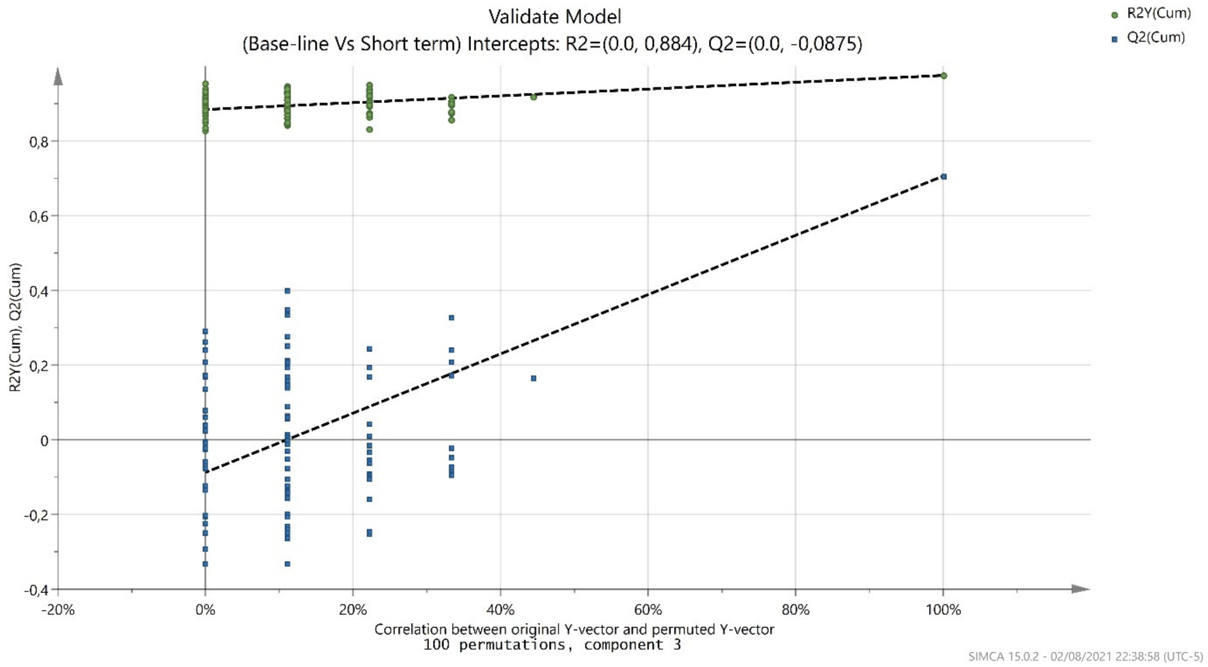

Figure S3: Results of PLS-DA model "base-line Versus Short Term" and permutation tests for its validation

Table S1. Baseline clinical characteristics of the volunteers

	**Mean ± DE**	**Min - Max**
Systolic blood pressure (mmHg)	120.6 ± 7.4	109.0 - 136.0
Diastolic blood pressure (mmHg)	76.1 ± 9.7	62.0 - 97.0
BMI (kg/m^2)	24.1 ± 1.9	19.9 -28.2
Glucose (mg/dL)	88.0 ± 6.3	76.0 -98.0
Glycosylated hemoglobin (%)	5.5 ± 0.2	5.0-6.0
Insulin (μIU/ml)	6.5 ± 3.0	1.8-11.6
HOMA IR	1.4 ± 0.7	0.4-2.5
Total cholesterol (mg/dL)	178.9 ± 22.7	126-223
HDL (mg/dL)	46.6 ± 8.5	36-65
LDL (mg/dL)	109.8 ± 21.4	77-146
Triglycerides (mg/dL)	112.9 ± 54.4	55-259
Blood creatinine (mg/dL)	1.0 ± 0.1	0.8-1.2
Urinary creatinine (mg/dL)	164 ± 57.5	83.8-276.8
AST (U/L)	21.3 ± 5.7	14.9-34.5
ALT (U/L)	21.2 ± 9.1	11.7-50.4
Age (years)	36.6 ± 7.4	27-49

ALT: Alanine Aminotransferase; AST: aspartate aminotransferase; BMI: body mass index; HDL: high-density lipoprotein; HOMA IR: insulin resistance index using the homeostasis model assessment; LDL: low-density lipoprotein.

Table S2: List of discriminant metabolites, significantly changed after acute nutritional intervention with *Physalis Peruviana* putatively identified with high confidence (Compounds written in bold correspond toTier 1 and in italic to *tier 2*)

Match	HMDB	PUBCHEM	KEGG	Average Base line	Average Acute	Fold change	P-value BvsA	VIP BvsST
**Uridine isomer**	HMDB0000296	6029	C00299	1,16	0,62	-1,88	4,76E-11	2,56
**Uridine**	HMDB0000296	6029	C00300	1,11	0,65	-1,71	7,69E-10	2,52
**Putrescine**	HMDB0001414	1045	C00134	1,05	0,53	-1,98	9,19E-08	2,34
**4-Hydroxyproline**	HMDB0000725	5810	C01157	1,02	0,81	-1,26	6,81E-07	1,44
**Ethanolamine**	HMDB0000149	700	C00189	1,05	0,86	-1,23	1,39E-06	1,80
N1-Acetylspermidine	HMDB0001276	496	C00612	1,15	0,79	-1,45	5,19E-06	1,95
**Citrulline**	HMDB0000904	9750	C00327	1,04	0,85	-1,23	5,69E-06	1,31
**Uracil**	HMDB0000300	1174	C00106	1,16	0,89	-1,31	9,58E-06	1,78
Isomer of Ethanolamine	HMDB0000149	700	C00189	1,09	0,90	-1,20	1,17E-05	1,75
**Citrulline**	HMDB0000904	9750	C00327	1,04	0,86	-1,20	1,83E-05	1,34
**L-Kynurenine**	HMDB0000684	161166	C00328	1,05	0,84	-1,24	2,19E-05	1,48
Diethanolamine	HMDB0004437	8113	C06772	0,87	1,31	1,51	2,83E-05	2,23
Syringic acid	HMDB0002085	10742	C10833	0,39	1,82	4,67	3,45E-05	2,05
Monodehydroascorbate	HMDB0000044	54670067	C01041	1,03	0,80	-1,28	4,00E-05	1,51
N(gamma)-Acetyldiaminobutyrate	HMDB0031411	902	C06442	0,85	1,06	1,25	4,93E-05	1,57
1-Methylguanosine	HMDB0001563	96373	C04545	0,99	0,78	-1,27	6,86E-05	1,82
Proline isomer	HMDB0000162	145742	C00148	0,90	1,11	1,22	1,07E-04	1,47
(R)-1-Aminopropan-2-ol	HMDB0012136	6071	C03194	1,04	1,28	1,24	1,29E-04	1,78
**Prolyl-Valine**	HMDB0029030	142984	N/A	1,29	1,64	1,27	1,59E-04	1,73
p-Aminobenzoic acid isomer	HMDB0001392	978	C00568	0,90	1,11	1,22	2,70E-04	1,40
**Proline**	HMDB0000162	145742	C00148	0,93	1,06	1,13	2,99E-04	1,50
p-Aminobenzoic acid isomer	HMDB0001392	978	D02456	0,91	1,10	1,21	3,03E-04	1,42
4,6-Dihydroxyquinoline	HMDB0004077	440738	C05639	1,35	1,12	-1,20	3,18E-04	1,57
**Adenosine**	HMDB0000050	60962	C00212	1,70	0,53	-3,20	3,32E-04	1,92
o-Hydroxylaminobenzoate	N/A	349497	C16235	1,04	0,84	-1,24	7,12E-04	1,88
L-3-Aminodihydro-2(3H)-furanone	HMDB0029387	73509	C02926	1,91	2,47	1,30	9,03E-04	1,72
5′-Deoxyadenosine	HMDB0001983	439182	C05198	0,86	1,28	1,49	1,01E-03	1,72
3-Hydroxy-L-proline	HMDB0002113	11137200	C04397	1,39	0,83	-1,67	1,03E-03	1,35
**Glycine**	HMDB0000123	750	C00037	1,04	0,89	-1,16	1,18E-03	1,24
**Leucyl-Glutamate**	HMDB0028928	5259589	N/A	0,90	1,09	1,21	1,45E-03	1,63
**Prolyl-Leucine**	N/A	173815	N/A	1,23	1,50	1,22	2,22E-03	1,51

4-Hydroxy-L-tryptophan	N/A	85479	N/A	1,01	0,84	-1,20	2,54E-03	1,17
3-Hydroxyanthranilic acid	HMDB0001476	86	C00632	0,70	1,15	1,65	3,50E-03	1,42
**Prolyl-phenylalanine**	HMDB0011179	6351946	N/A	1,15	1,32	1,15	3,70E-03	1,27
**5-Hydroxylysine**	HMDB0000450	3032849	C16741	0,86	1,07	1,25	4,37E-03	1,67
**Symmetric dimethylarginine**	HMDB0003334	169148	N/A	1,01	0,90	-1,12	4,37E-03	1,21
Cysteine	HMDB0000574	5862	C00097	1,24	0,97	-1,27	4,63E-03	1,25
**Aminoadipic acid**	HMDB00510	92136	C00956	0,81	1,05	1,29	5,15E-03	1,48
Biopterin	HMDB0000468	445040	C06313	1,00	1,48	1,48	5,48E-03	1,40
N-Acetylindoxyl	N/A	5353	C02298	0,82	1,23	1,51	5,74E-03	1,23
**prolyl-proline**	HMDB0011180	11622593	N/A	1,23	1,58	1,28	7,45E-03	1,31
**3-Hydroxyphenylacetic acid**	HMDB00440	12122	C05593	0,85	1,00	1,18	8,18E-03	1,60
**Aminoadipic acid isomer**	HMDB00510	92136	C00956	0,80	1,05	1,32	8,97E-03	1,46
Cystein isomer	HMDB0000574	5862	C00097	1,03	0,84	-1,23	1,01E-02	1,33
**Thymine**	HMDB0000262	1135	C00178	0,92	0,75	-1,23	1,21E-02	1,27
**Prolyl-Asparagine**	HMDB0029012	18464218	N/A	1,17	1,36	1,17	1,23E-02	1,21
**Saccharopine**	HMDB0000279	160556	C00449	0,82	1,14	1,40	1,51E-02	1,43
5-Aminopentanal	HMDB0012815	443849	C12455	1,08	0,98	-1,10	1,53E-02	1,25
**Cystine**	HMDB0000192	67678	C00491	1,03	0,84	-1,23	2,00E-02	1,26
4-Hydroxyphenylglyoxylate	N/A	6381	C03590	0,93	1,08	1,16	2,32E-02	1,33
Diaminopimelic acid	HMDB0001370	439283	C00666	1,02	1,18	1,15	2,48E-02	1,26
**Ribothymidine**	HMDB0000884	445408	N/A	1,04	0,94	-1,10	2,74E-02	1,25
L-Cystine	HMDB0000192	67678	C00491	1,04	0,83	-1,24	3,34E-02	1,32
**5'-Methylthioadenosine**	HMDB0001173	439176	C00170	0,76	0,91	1,19	3,58E-02	1,23
**5-Aminolevulinic acid**	HMDB01149	137	C00430	1,00	1,13	1,12	4,86E-02	1,39

Table S3: List of List of discriminant metabolites significantly changed after short-term nutritional intervention with *Physalis Peruviana* putatively identified with high confidence (Compounds written in bold correspond to Tier 1 and in italic to *tier 2*)

Match	HMDB	PUBCHEM	KEGG	Average Base line	Average Acute	Fold change	P-value BvsA	VIP BvsST
3,4-Dihydroxymandelaldehyde	HMDB0006242	151725	C05577	1,21	0,89		7,18E-05	2,29
4-hydroxymethylsalicylic acid	N/A	656883	N/A	1,21	0,89	Fold change	6,35E-05	2,26
**Adenosine**	HMDB0000050	60962	C00212	1,66	0,56	-1,36	1,96E-04	2,17
L-3-Aminodihydro-2(3H)-furanone	HMDB0029387	73509	C02926	1,91	2,66	-1,36	2,19E-04	2,13
Quinoline-4,8-diol	HMDB0060289	440737	C05637	1,35	1,13	-2,99	1,43E-06	2,06
3,4-Dihydroxymandelic acid	HMDB0001866	85782	C05580	0,90	0,75	1,39	4,51E-03	2,03
**4-Hydroxybenzoic acid**	HMDB0000500	135	C00156	1,13	0,93	-1,20	4,57E-05	1,91
2-Hydroxy-2,4-pentadienoate	HMDB0037196	62166	C00596	0,93	1,34	-1,19	1,56E-04	1,87
p-Aminobenzoic acid isomer	HMDB0001392	978	D02456	0,83	0,99	-1,22	2,18E-03	1,85
**5'-Methylthioadenosine**	HMDB0001173	439176	C00171	0,76	1,04	1,43	7,71E-04	1,74
L-Lysine	HMDB0000182	5962	C00047	0,85	1,04	1,19	5,05E-04	1,70
6-Amino-2-oxohexanoate	HMBD12151	439954	C03239	0,95	1,12	1,37	8,22E-04	1,69
Dihydroxyfumaric acid	HMDB02050	8618	C00975	1,09	0,97	1,22	2,25E-03	1,60
**Aminoadipic acid isomer**	HMDB00510	92136	C00956	0,80	1,02	1,18	3,55E-03	1,58
Aminoadipic acid	HMDB00510	92136	C00956	0,71	0,99	-1,12	5,27E-03	1,58
N(gamma)-Acetyldiaminobutyrate	HMDB0031411	902	C06442	0,85	0,97	1,27	2,11E-02	1,54
**Aminoadipic acid**	HMDB00510	92136	C00956	0,81	1,00	1,41	3,99E-03	1,52
Spermidine	HMDB0001257	1102	C00315	0,95	1,39	1,14	8,87E-04	1,52
7-Aminomethyl-7-carbaguanine	HMDB0011690	171	C16675	0,80	1,15	1,23	2,90E-03	1,50
Biopterin isomer	HMDB0000468	445040	C06313	0,89	1,16	1,46	7,81E-03	1,47
Biopterin isomer	HMDB0000468	445040	C06313	0,91	1,17	1,43	1,66E-02	1,46
O-Acetyl-L-homoserine	HMDB0029423	528	N/A	0,73	0,97	1,30	1,43E-02	1,44
Coniferyl acetate	N/A	12835430	N/A	0,95	0,78	1,29	1,44E-02	1,43
5′-Deoxyadenosine	**HMDB0001983**	**439182**	**C05198**	0,86	1,03	1,34	4,86E-03	1,40
Isomer of 2′-O-Methyladenosine	HMDB04326	317398	C04779	0,85	1,18	-1,23	8,01E-03	1,39
2-Hydroxymuconate semialdehyde	N/A	5280366	N/A	0,74	0,91	1,20	1,78E-02	1,38
**Saccharopine**	HMDB0000279	160556	C00449	0,82	1,00	1,39	1,45E-02	1,37

**Homoserine**	HMDB0000719	12647	C00263	0,90	1,12	1,23	8,01E-03	1,36
Aminohydroquinone	N/A	3017497	N/A	0,75	0,97	1,22	2,33E-02	1,35
N-Acetylindoxyl	N/A	439701	N/A	0,82	1,40	1,25	5,13E-03	1,34
**3-Hydroxymandelic acid**	HMDB0000750	86957	N/A	1,17	1,06	1,29	2,91E-02	1,33
3-Hydroxyanthranilate	HMDB01476	86	C00632	0,70	1,25	1,72	5,16E-03	1,33
isomer of 4-aminobutanal	HMDB0001080	118	C02903	1,06	0,92	-1,10	1,35E-02	1,33
5-Aminopentanamide	HMDB12176	439358	C00990	0,87	1,15	1,80	6,20E-03	1,33
cis,cis-4-Hydroxymuconic semialdehyde	N/A	9543170	N/A	0,84	1,09	-1,15	3,08E-02	1,32
4-Aminobutyraldehyde	HMDB0001080	118	C02903	1,04	0,90	1,33	9,53E-03	1,32
Isomerof 4-Aminobutyraldehyde	HMDB0001080	118	C02903	1,04	0,89	1,31	1,60E-02	1,31
**Valyl-Lysine**	HMDB29132	168058	N/A	1,19	0,93	-1,16	1,72E-02	1,31
Isomer of 4-Aminobutyraldehyde	HMDB0001080	118	C02903	1,01	0,89	-1,16	1,04E-02	1,29
**(S)-4-Amino-5-oxopentanoate**	N/A	16722117	N/A	1,28	1,09	-1,28	1,00E-02	1,23
**Ethanolamine**	HMDB0000149	700	C00189	1,05	0,94	-1,14	3,19E-03	1,22
